# Finding the C_4_ sweet spot: cellular compartmentation of carbohydrate metabolism in C_4_ photosynthesis

**DOI:** 10.1093/jxb/erab290

**Published:** 2021-06-17

**Authors:** Robert T Furbank, Steven Kelly

**Affiliations:** 1 ARC Centre of Excellence for Translational Photosynthesis, Research School of Biology, The Australian National University, Canberra, ACT 2601, Australia; 2 Department of Plant Sciences, University of Oxford, South Parks Road, Oxford OX1 3RB, UK; 3 Bielefeld University, Germany

**Keywords:** Bundle sheath cells, C_4_ photosynthesis, mesophyll cells, starch, sucrose

## Abstract

The two-cell type C_4_ photosynthetic pathway requires both anatomical and biochemical specialization to achieve a functional CO_2_-concentrating mechanism. While a great deal of research has been done on Kranz anatomy and cell-specific expression and activity of enzymes in the C_4_ pathway, less attention has been paid to partitioning of carbohydrate synthesis between the cell types of C_4_ leaves. As early as the 1970s it became apparent that, in the small number of species examined at the time, sucrose was predominantly synthesized in the mesophyll cells and starch in the bundle sheath cells. Here we discuss how this partitioning is achieved in C_4_ plants and explore whether this is a consequence of C_4_ metabolism or indeed a requirement for its evolution and efficient operation.

## Teaser

C_4_ plants partition starch and sucrose biosynthesis between bundle sheath and mesophyll cells. Here we review why and how this is achieved, and discuss the relevance for C_4_ photosynthesis.

## Introduction

### Metabolic specialization in C_4_ photosynthesis

**Fig. 1.**
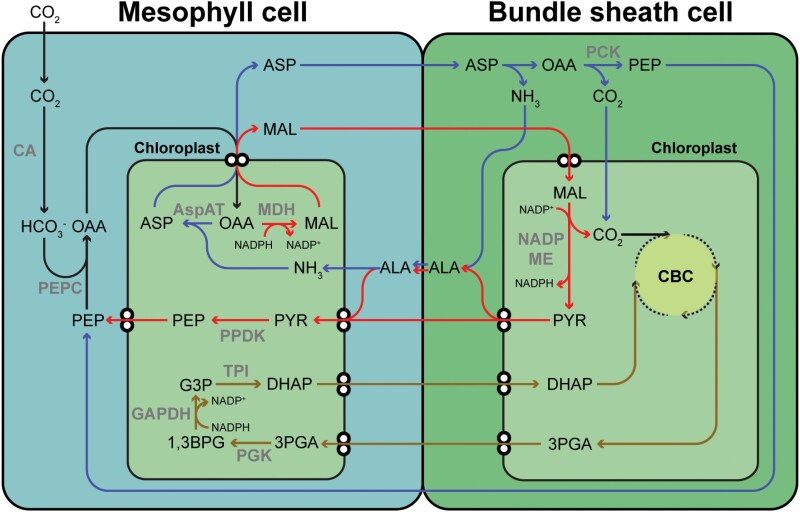
Diagram of a hybrid NADP-ME and PCK C_4_ cycle. The direction of flux through the NADP-ME component of the cycle is shown by red arrows, the direction of flux through the PCK component of the cycle is shown by blue arrows, and routes common to both pathways are shown by black arrows. The direction of flux through the triose phosphate shunt is shown by brown arrows. Reactions on these pathways that consume or regenerate NADPH are shown. CA, carbonic anhydrase; PEPC, phosphoenolpyruvate carboxylase; MDH, NADP-malate dehydrogenase; AspAT, aspartate amino transferase; PCK, phosphoenolpyruvate carboxykinase; NADP-ME, NADP-malic enzyme; PPDK, pyruvate, phosphate dikinase; PGK, phosphoglycerate kinase; GAPDH, glyceraldehyde 3-phosphate dehydrogenase; TPI, triose phosphate isomerase; CBC, Calvin–Benson cycle; PEP, phosphoenolpyruvate; OAA, oxaloacetate; MAL, malate; ASP, aspartate; ALA, alanine; 3PGA, 3-phosphoglycerate; 1,3BPG, 1,3-bisphosphoglycerate; G3P, glyceraldehyde 3-phosphate; DHAP, dihydroxyacetone phosphate.

Most plant species carry out all of the reactions required to harvest light energy and fix carbon within a single cell. However, some plants known as C_4_ plants have evolved a way to partition these reactions between two separate cells. The operation of the two-cell or ‘Kranz’ C_4_ photosynthetic pathway requires a combination of anatomical and biochemical specialization to separate the initial fixation of atmospheric CO_2_ from the site of CO_2_ release and concentration (reviewed in Hatch, 1987; Sage *et al.*, 2012; Furbank, 2016). Typically, this separation occurs between the mesophyll cells and bundle sheath cells in the leaf, and a simplified diagrammatic representation of the C_4_ pathway and associated metabolism from *Zea mays* is shown in Fig. 1. Remarkably, this partitioning of photosynthesis between two cells evolved independently at least 60 times in 19 different families of angiosperms (Sage, 2016), and consequently it is often cited as one of the most remarkable examples of convergent evolution in eukaryotes.

Perhaps unsurprisingly given the repeated evolutionary origins, C_4_ species exhibit substantial metabolic, anatomic, and phenotypic diversity. Accordingly, C_4_ species are conventionally categorized into one of three metabolic subtypes [NADP-malic enzyme (NADP-ME), NAD-malic enzyme (NAD-ME), and phosphoenolpyruvate carboxykinase (PCK)] according to the expression and activity of the decarboxylase present in the bundle sheath. However, it is now known that the division of these decarboxylation types is not invariant, with some species showing mixtures of these mechanisms (Furbank, 2011; Y. Wang *et al.*, 2014; von Caemmerer and Furbank, 2016). The complexity of the C_4_ pathway is further increased by the existence of multiple different metabolite transport mechanisms (Aoki *et al.*, 1992), and dimorphic chloroplasts in the NADP-ME-type C_4_ species, where in many cases PSII is reduced or almost absent in the bundle sheath cell chloroplasts (Woo *et al.*, 1970; Munekage and Taniguchi, 2016). While NADP-ME in the bundle sheath chloroplasts of these species can generate some of the NADPH required to support the photosynthetic carbon reduction cycle via malate decarboxylation, in the absence of whole-chain electron transport at least half of the 3-phophoglycerate (3PGA) must return to the mesophyll chloroplasts for reduction (Hatch, 1987; von Caemmerer and Furbank, 2016). It is thought that this return of 3PGA to the mesophyll cell provides some flexibility in the split of ATP production and reducing power between the cell types even in NAD-ME- and PCK-type plants (Hatch, 1987; von Caemmerer and Furbank, 2016, and references therein). In the mesophyll cell chloroplast, it has been shown that the C_4_ and C_3_ pathways can also share carbon flux by the interchange of phosphoenolpyruvate (PEP) and 3PGA via the phosphoglycerate mutase and enolase pathway (Furbank and Leegood, 1984; von Caemmerer and Furbank, 2016). The implications of this partitioning of triose phosphates between the mesophyll cells and bundle sheath cells for regulation of sucrose and starch synthesis are largely unexplored.

## Sucrose and starch synthesis in leaves of C_4_ plants

Long before the discovery of the C_4_ pathway, Kranz anatomy had been observed in tropical grasses (Haberlandt, 1904). Some decades later came the curious observation that starch was present almost exclusively in bundle sheath cells of maize leaves under standard growth conditions (Rhoades and Cavalho, 1944). Their work on variegated maize mutants and exogenous sugar application suggested that the sugar substrates were imported to the bundle sheath from the mesophyll and that addition of exogenous sucrose could overcome this strict cellular partitioning. Subsequent work (reviewed in Laetsch, 1974) explored this in more detail and, following the discovery of the C_4_ pathway, it was proposed that in maize, sucrose was made exclusively in the mesophyll cells and starch was localized to the bundle sheath cells under standard growth conditions (Downton and Hawker, 1973). In this work, the authors used mechanical grinding of maize leaves to separate bundle sheath strands from mesophyll cell contents and assayed each fraction for the enzymes of sucrose and starch metabolism (for reviews on the metabolic pathways of sucrose and starch metabolism and differences between C_3_ species, see Stitt and Zeeman, 2012; Smith and Zeeman, 2020). Despite the challenges of obtaining uncontaminated cell separations in this early research, activities of ADP glucose pyrophosphorylase and starch synthase were much lower in mesophyll cells compared with bundle sheath strand preparations. Moreover, the bulk of invertase, sucrose phosphate synthase, sucrose phosphate phosphatase, and UDP glucose pyrophosphorylase activities was found in mesophyll fractions. Interestingly, the authors observed that exposure of maize leaves to continuous illumination for 2.5 days caused the starch content of mesophyll cells to increase greatly, suggesting some plasticity in this partitioning of starch metabolism. Despite these remarkable observations, the authors concluded that the cell-specific partitioning of enzyme activity was not sufficient to explain the partitioning of sucrose and starch synthesis observed, and proposed that sugar supply was potentially more important in apportioning flux in the two compartments.

Subsequent analysis of maize leaves along the linear developmental axis of the leaf blade revealed that the extent of accumulation of starch and sucrose in bundle sheath cells differed along the leaf blade, from almost absent at the base of the leaf to ‘massive’ accumulation towards the tip (Majeran *et al.*, 2010). Moreover, partitioning of sucrose and starch synthesis was further examined as protocols for protoplast isolation from C_4_ leaves became more commonplace, with reliable isolated mesophyll protoplast and cell preparation with low bundle sheath cell contamination (Mbaku *et al.*, 1978; Usuda and Edwards, 1980). In the C_4_ species *Panicum miliaceum* (NAD-ME type) and *Brachiaria eruciformis* (PCK type), the distribution of biosynthetic enzymes was not as clear as in maize (Usuda and Edwards, 1980), while in *Digitaria penzii* (NADP-ME type), enzymes of sucrose synthesis appeared to be present in both compartments, but newly fixed carbon appeared to be partitioned primarily to starch in the bundle sheath cells (Mbaku *et al.*, 1978). Wider investigation into the partitioning of sucrose and starch synthesis across C_4_ angiosperms revealed that localization of starch biosynthesis in bundle sheath cells was common irrespective of C_4_ origin, or anatomical/biochemical subtype (Edwards and Voznesenskaya, 2010). Moreover, even in single-cell C_4_ species (i.e. C_4_ species that partition the reactions of photosynthesis between two chloroplast types within the same cell) it became apparent that selective synthesis of starch occurred only in one chloroplast subtype (Edwards and Voznesenskaya, 2010; Voznesenskaya *et al.*, 2013). Thus, partitioning of sucrose and starch biosynthesis appears to be a fundamental part of C_4_ photosynthetic partitioning.

The hypothesis of Downton and Hawker (1973) that in maize sucrose and starch syntheses were physically separated between the cell types was borne out by labelling and enzyme localization experiments of Furbank *et al.* (1985). This work used a ‘leaf rolling’ rapid kill cell separation system (Leegood, 1985) which squeezed out leaf mesophyll contents after ^14^CO_2_ labelling with little contamination from the bundle sheath cells. These experiments clearly showed that >80% of newly fixed carbon was partitioned into sucrose in the mesophyll cells and no starch was detected in the mesophyll fraction. The enzymes sucrose phosphate synthase and cytosolic fructose-1,6-bisphosphatase, required for sucrose biosynthesis, were located predominantly in the mesophyll cells (Furbank *et al.*, 1985). Starch synthesized in the bundle sheath must be broken down at night and converted to sucrose for export from the leaf. This presumably must require the presence of sucrose phosphate synthase in the bundle sheath cells. Sucrose phosphate synthase activity, and the ability to synthesize sucrose from radiolabelled fructose-6-phosphate, has been demonstrated in isolated bundle sheath cells of the C_4_ grasses *Panicum miliaceum* (NAD-ME) and *Sorghum bicolor* (NADP-ME), and the C_4_ NADP-ME dicot *Flaveria bidentis* (Lunn *et al.*, 1997). In a survey of C_4_ species, Lunn and Furbank (1997) showed that in *Zea mays* (NADP-ME and PCK) and *Atriplex spongiosa* (NAD-ME), sucrose phosphate synthase was located almost exclusively in the mesophyll cells. However, in other species, sucrose phosphate synthase was found in both cell types, with the activity in the bundle sheath cells ranging from 5% of the total leaf activity in *Echinochloa crus-galli* (NADP-ME grass) to 35% in *S. bicolor*. This suggests that while sucrose synthesis is preferentially localized to the mesophyll in maize, a substantial capacity for bundle sheath sucrose synthesis exists, and that relative rates are variable among C_4_ plants with little correlation with decarboxylation type.

While the site of sucrose synthesis in C_4_ leaves may differ greatly between species, there is considerable evidence that under standard growth conditions, starch is made exclusively in bundle sheath cells of all C_4_ leaves surveyed (Lunn and Furbank, 1997, 1999). Potassium iodide staining at the end of the photoperiod detected no starch in the mesophyll cells and strong signal in the bundle sheath compartment of leaves of 11 species spanning all three decarboxylation types grown at a peak irradiance of 2000 μmol m^–2^ s^–1^ (Lunn and Furbank, 1997). This was a surprising result, given the diversity between species in the partitioning of newly fixed carbon between sucrose and starch, and the ratio of sucrose to starch detected at the end of the photoperiod across C_4_ species (Lunn and Hatch, 1995). The ratio of sucrose to starch accumulated during the day in this study varied from 0.02 in *Flaveria trinervia* (NADP-ME dicot) to 1.03 in *Eleusine indica* (NAD-ME grass). Such large differences between species in strategies for partitioning carbon to temporary storage carbohydrates but exclusive partitioning of starch to the bundle sheath cells raises questions as to the mechanisms controlling this process.

## Transcriptional regulation of sucrose and starch synthesis in leaves of C_4_ plants

**Fig. 2.**
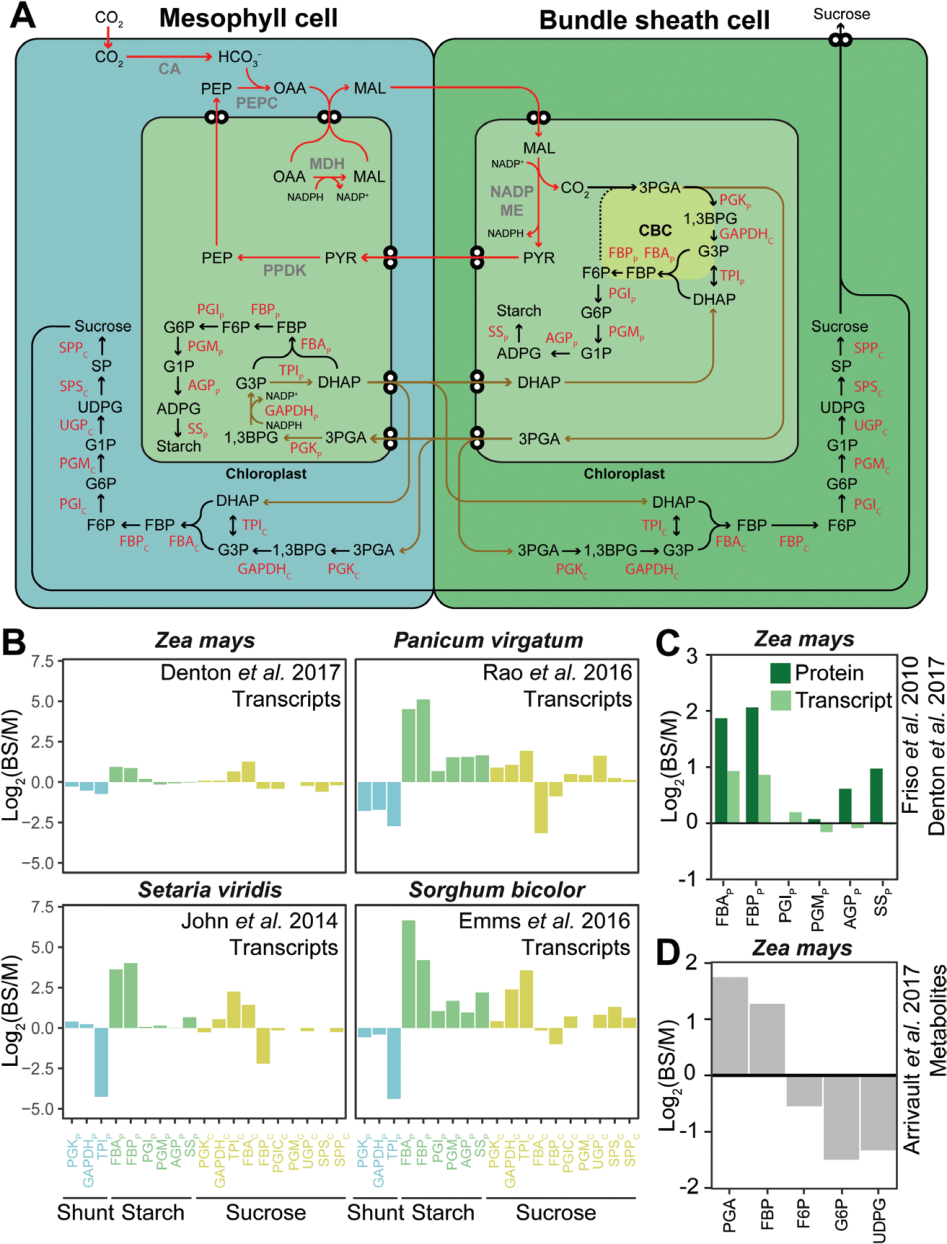
Overview of starch and sucrose metabolism in C_4_ leaves inferred from published cell type-specific transcriptome, proteome, and metabolome data. (A) A cartoon diagram of NADP-ME C_4_ photosynthesis with pathways for starch and sucrose biosynthesis shown in each cell type. (B) Abundance data for transcripts encoding starch and sucrose biosynthesis genes from (A). Transcripts encoding each enzyme were summed to provide a single value for each enzyme; individual transcript data and transcript to enzyme mappings can be found in Table S1 at Dryad Digital Repository https://doi.org/10.5061/dryad.cz8w9gj3v. (C) Comparison between cell type-specific transcript and protein accumulation. (D) Patterns of metabolite accumulation. In all cases, the species name and citation for the original data are provided in the subpanel of the figure. For (A–C) subscript ‘C’ indicates cytosolic, and subscript ‘P’ indicates plastidic. PCK, phosphoglycerate kinase; GAPDH, glyceraldehyde 3-phosphate dehydrogenase; TPI, triose phosphate isomerase; FBA, fructose-1,6-bisphosphate aldolase; FBP, fructose-1,6-bisphosphatase; PGI, phosphoglucose isomerase; PGM, phosphoglucomutase; AGP, ADP-glucose pyrophosphorylase; SS, starch synthase; UGP, UDP-glucose pyrophosphorylase; SPS, sucrose phosphate synthase; SPP, sucrose phosphate phosphatase. For (D), PGA, phosphoglyceric acid; FBP, fructose-1,6-bisphosphate; F6P, fructose-6-phosphate; G6P, glucose-6-phophate; UDPG, UDP-glucose. See legend to Fig. 1 for metabolite names.

Given that the chloroplasts of the bundle sheath cells are the site of the Calvin–Benson–Bassham cycle, and thus the site of fructose-6-phosphate biosynthesis, it is perhaps most parsimonious for the plant to accumulate all other enzymes of the starch biosynthesis pathway in this cell type and thus localize starch biosynthesis to the bundle sheath. Although the availability of transcriptome and proteome data to assess this hypothesis is somewhat limited, there are four C_4_ species that have been subject to cell type-specific transcriptome profiling: *S. bicolor* (NADP-ME; Emms *et al.*, 2016); *Setaria viridis* (NADP-ME; John *et al.*, 2014), *Panicum virgatum* (NAD-ME; Rao *et al.*, 2016), and *Z. mays* (NADP-ME and PCK; Denton *et al.* 2017). Thus, for these species, it is possible to assess whether the genes encoding the enzymes of the core starch and sucrose biosynthesis pathways exhibit cell type preferential expression that may help explain the patterns of starch accumulation (Fig. 2A). With the exception of *Z. mays*, transcripts encoding all enzymes in starch biosynthesis accumulate preferentially in bundle sheath cells (Fig. 2B). Interestingly, although the transcripts encoding phosphoglucomutase, ADP-glucose pyrophosphorylase, and starch synthase accumulate preferentially in mesophyll cells in maize, their encoded protein products accumulate preferentially in bundle sheath cell chloroplasts (Fig. 2C). Thus, it is likely that all enzymes involved in starch biosynthesis preferentially accumulate in the bundle sheath cells of these C_4_ plants.

In contrast, the expression of genes in the sucrose biosynthesis pathway did not show a clear cell type association (Fig. 2B). Interestingly, transcripts encoding cytosolic isoforms of phosphoglycerate kinase, glyceraldehyde 3-phosphate dehydrogenase, and triose phosphate isomerase generally accumulated preferentially in the bundle sheath of these C_4_ plants. Only transcripts encoding cytosolic fructose-1,6-bisphosphatase accumulated preferentially in mesophyll cells in all four species. Transcripts encoding all other sucrose biosynthesis enzymes exhibited contrasting patterns of cell type preferential accumulation (Fig. 2B). In general, transcripts in *S. bicolor* and *P. virgatum* accumulated preferentially in the bundle sheath, while transcripts in *Z. mays* and *S. viridis* accumulated preferentially in the mesophyll (Fig. 2B). The pattern of transcript accumulation in maize is also mirrored in the accumulation of intermediate metabolites in *Z. mays* (Arrivault *et al.*, 2017; Fig. 2D). Thus, while there is a consistent transcriptome signal for starch biosynthesis in the bundle sheath, the transcriptome signature of sucrose biosynthesis shows considerable variation between different C_4_ species.

**Fig. 3.**
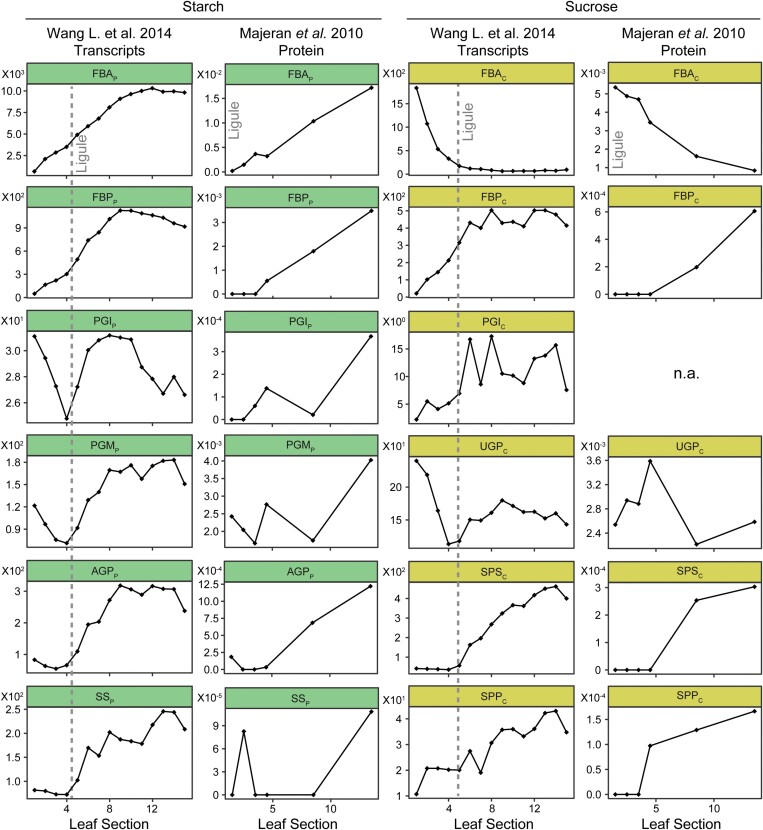
Patterns of transcript and protein accumulation along the leaf blade in *Zea mays*. Sections 1–4 in the transcriptome datasets correspond to leaf sheath tissue, and sections 5–15 correspond to leaf blade. The position of the ligule (and thus the transition from leaf sheath to leaf blade) is indicated by the vertical dashed line. All protein samples were sampled above the ligule, and the ligule is marked as the position where *y*=0. Transcripts encoding each enzyme were summed to provide a single value for each enzyme. Similarly, protein isoforms corresponding to the same enzyme activity in the same subcellular location were summed. Individual transcript and protein abundance data and transcript to enzyme mappings can be found in Table S1 at Dryad. FBA_P_: plastidic fructose-1,6-bisphosphate aldolase; FBA_C_, cytosolic fructose-1,6-bisphosphate aldolase; FBP_P_, plastidic fructose-1,6-bisphosphatase; FBP_C_, cytosolic fructose-1,6-bisphosphatase; PGI_P_, plastidic phosphoglucose isomerase; PGI_C_, cytosolic phosphoglucose isomerase; PGM_P_, plastidic phosphoglucomutase; AGP_P_, plastidic ADP-glucose pyrophosphorylase; SS_P_, plastidic starch synthase; UGP_C_, cytosolic UDP-glucose pyrophosphorylase; SPS_C_, cytosolic sucrose phosphate synthase; SPP_C_, cytosolic sucrose phosphate phosphatase.

A cautionary note for the interpretation of these transcriptome data comes from the highly dynamic nature of transcript abundance along a grass leaf blade. For example, there are striking differences in the patterns of accumulation of transcripts and mass spectra corresponding to enzymes of starch and sucrose biosynthesis along the length of the leaf blade (Majeran *et al.*, 2010; L. Wang *et al.*, 2014; Fig. 3). For example, with the exception of phosphoglucose isomerase, transcripts and mass spectra corresponding to plastid-localized enzymes of starch biosynthesis generally increase in abundance along the length of the leaf blade (Fig. 3). In contrast, transcripts and mass spectra corresponding to cytosolic-localized fructose-1,6-bisphosphate aldolase decrease along the axis of the leaf blade while other enzymes of sucrose biosynthesis either exhibit no change or increase in abundance (Fig. 3). Thus, the entry points to the two pathways show opposite patterns of accumulation, and the patterns of transcript and protein accumulation mirror the accumulation of starch along the maize leaf (Majeran *et al.*, 2010). This strong linear dependency further complicates the partitioning of starch and sucrose between the bundle sheath and the mesophyll as the age and position along the leaf are likely to alter the results obtained and thus the interpretations that are made.

## Control of sucrose and starch synthesis and efficient operation of the C_4_ mechanism

In early work on carbon partitioning between bundle sheath and mesophyll cells of C_4_ leaves, enzyme activity-based localization studies formed the basis for most of the available information (e.g. Downton and Hawker, 1973; Mbaku *et al.*, 1978; Usuda and Edwards, 1980), supplemented by some rapid cell separation and radiolabelling studies (Furbank *et al.*, 1985; Stitt and Heldt, 1985). As many of the early studies did not use immunological quantification of enzyme levels, it is possible that there may be poor correspondence between activity and the protein/transcript present in particular cell types. It is also possible that regulation or properties of the C_4_ enzyme isoforms provide the basis for preferential partitioning of fixed carbon to starch in the bundle sheath or sucrose in the mesophyll cells. Metabolic regulation of sucrose and starch synthesis has been extensively studied for more than half a century (Preiss, 1982; Winter and Huber, 2000) and there is evidence for altered kinetic properties of key enzymes in C_4_ leaves when compared with their C_3_ counterparts (e.g. Stitt and Heldt, 1985; Winter and Huber, 2000).

Perhaps the most important ramification for diurnal carbohydrate synthesis on C_4_ mechanism is the potential for a collapse in the metabolite gradient required to support the 3PGA/triose phosphate shuttle. The potential for sucrose synthesis in the mesophyll cells of maize to consume triose phosphates and collapse the gradient of triose phosphate required for diffusion back to the bundle sheath and regeneration of ribulose 1,5-bisphosphate (RuBP) was identified >30 years ago (Furbank *et al.*, 1985; Stitt and Heldt, 1985). It has been calculated that to support the return of reduced carbon to the bundle sheath chloroplast, a gradient of 10 mM triose phosphate would be required (Hatch and Osmond, 1976), a figure borne out from rapid fractionation of metabolites from maize mesophyll and bundle sheath cells (Leegood, 1985; Stitt and Heldt, 1985). Stitt and Heldt (1985) showed that the cytosolic fructose-1,6-bisphosphatase from maize has a lower affinity for its substrate fructose-1,6-bisphosphate than the C_3_ enzyme, particularly in the presence of the inhibitor fructose-2,6-bisphosphate which was shown to be predominantly in the mesophyll cells. In the presence of fructose-1,6-bisphosphate, aldolase, fructose-1,6-bisphosphate, and triose phosphate levels are in equilibrium, so that a decrease in affinity of fructose-1,6-bisphosphate would allow a higher concentration of triose phosphate to be maintained in mesophyll cells to support the diffusion gradient. These authors also proposed that this low-affinity form of cytosolic fructose-1,6-bisphosphatase may only be present in species where the 3PGA ‘shunt’ is obligatory due to the absence of PSII from the bundle sheath chloroplasts.

The 3PGA shuttle also adds complexity to the well-established regulation of starch biosynthesis in C_3_ leaves via allosteric regulation of ADP-glucose pyrophosphorylase (Preiss, 1982; Winter and Huber, 2000). This enzyme is a key control point in carbon partitioning between sucrose and starch via allosteric regulation by the relative levels of 3PGA (an activator of the enzyme) and Pi (an inhibitor). In a C_3_ chloroplast, triose phosphate is exported by counter-exchange for Pi (Fliege *et al.*, 1978) released during sucrose synthesis in the cytosol. If sucrose synthesis slows, Pi return is reduced, and 3PGA and triose phosphate will build up, activating starch biosynthesis (Herold, 1980; Preiss, 1982). In the case of bundle sheath cell chloroplasts, if 3PGA is exported in exchange for triose phosphate import rather than Pi, this fine allosteric control must operate differently. Indeed, if sucrose biosynthesis occurs primarily in the mesophyll, then Pi must return back to the bundle sheath chloroplast to support photophosphorylation. Moreover, the stoichiometries of Pi and triose phosphate exchange with 3PGA would need to be balanced to accommodate the regenerative phase of the Calvin–Benson–Bassham cycle, export of carbon for sucrose biosynthesis, and supply of Pi for photophosphorylation. The complexities of this regulatory network in C_4_ leaves have not been extensively studied.

The transcriptional data shown above for maize provide very little evidence that strict control of the site of sucrose and starch synthesis is achieved at the gene expression level. For example, the abundance of transcripts encoding starch biosynthesis genes is on average only ~3.3-fold higher in the bundle sheath than in the mesophyll, while the abundance of transcripts encoding sucrose biosynthesis genes is approximately the same in bundle sheath and mesophyll cells. It may be that regulation such as that proposed in these early studies plays a more important role in some C_4_ species in determining partitioning of stored carbon between cell types. This is also consistent with early observations that carbon excess conditions such as continuous illumination or removal of sinks can result in starch accumulation in both cell types of maize (Downton and Hawker, 1973).

## Is cell-specific location of carbohydrate synthesis a prerequisite for or a consequence of C_4_ evolution?

It has recently been suggested that the vascular differentiation of vein tissue which results in Kranz anatomy may be a result of the extension of the ‘starch sheath’ endodermis present in roots and stems of all angiosperms into the leaf blade in C_4_ plants (Slewinski, 2013). This hypothesis is based on the observation that the SCARECROW–SHORTROOT pathway of regulating radial vascular differentiation appears to be shared between root endodermis and bundle sheath tissues (Slewinski, 2013; Thomas *et al.*, 2019). These authors propose that in C_4_ leaves, bundle sheath cells may be ‘photosynthetic starch sheath’ cells such as those found in petioles and stems (Hibberd and Quick, 2002), and points out that starch sheath amyloplasts also have agranal chloroplasts (Langdale, 2011). This concept is further explored in grasses by Miyake (2016) who points out that the bundle sheath cells of rice appear to be specialized for starch storage, but only at the base of the leaf, and that this function ceases when the leaf blade becomes a photosynthetic source of carbon. Like the leaf sheath parenchyma cells, these cells appear to be a source of carbohydrate for remobilization, in this case during leaf development rather than during grain filling. In contrast, in many C_4_ leaves, early transitory starch storage occurs at the leaf base in both cell types then retreats mostly to the bundle sheath chloroplasts, although some C_4_ grasses accumulate mostly soluble sugars throughout development (Miyake and Maeda, 1978).

The primary location of sucrose biosynthesis in the mesophyll cells of many C_4_ species (Lunn and Furbank, 1997) presents an interesting challenge for sugar export to the phloem, as mesophyll cells are physically separated from the phloem by the bundle sheath. Accordingly, it has been proposed that apoplastic phloem loading may be a prerequisite for C_4_ evolution (Emms *et al.*, 2016). The authors propose that this enabled bundle sheath cells to have reduced symplastic connectivity to the phloem with enhanced connectivity to the mesophyll, thus, supporting diffusion of C_4_ cycle metabolites between the specialized cell types, minimizing leakage of these same metabolites to the phloem, and reducing the soluble sugar content of the bundle sheath which would act to inhibit photosynthesis. This hypothesis may also be supported by the almost universal paradigm of starch storage preferentially occurring in the bundle sheath compartment of C_4_ leaves as this would serve to further reduce bundle sheath cell sugars by removing the need for soluble sugars to be stored during the diurnal cycle. However, in the NADP-ME-type species *Z. mays* (Bezrutczyk *et al.*, 2018), *S. bicolor* (Emms *et al.*, 2016), and *S. viridis* (Chen, 2021), SWEET13, the plasma membrane sucrose effluxer protein shown to be involved in phloem loading in maize (Bezrutczyk *et al.*, 2018), is almost exclusively expressed in the bundle sheath cells. If sucrose is almost exclusively synthesized in the mesophyll cells of these species (Furbank *et al.*, 1985; Lunn and Furbank, 1995), then a gradient in sucrose between mesophyll and bundle sheath cells would be required to support export of carbon to the phloem. There are currently no data on sugar levels in the mesophyll cells relative to bundle sheath cells, but these would shed more light on how this important process functions and its relevance to the evolution of C_4_ plants.

At present, there is a paucity of studies on the location of starch and sucrose biosynthetic enzymes or their corresponding transcripts in the bundle sheath and mesophyll cells of diverse C_4_ species. Hence it is not possible to carry out a comprehensive phylogenetic analysis across multiple origins of C_4_ evolution to determine whether partitioning of starch synthesis to bundle sheath cells is a pre-condition for C_4_ evolution. Undoubtedly, advances in molecular imaging technologies such as MS imaging and infrared microscopy (Buchberger *et al.*, 2018), as well as advances in leaf cell separation/fractionation technologies coupled with a reduction in the cost of cell-specific transcriptomes, proteomes, and metabolomes, will help resolve this question.

## Data Availability

Data on individual transcript abundance and transcript to enzyme mappings, and other quantitative data associated with the figures presented in this review are available at Dryad Digital Repository https://doi.org/10.5061/dryad.cz8w9gj3v; Furbank and Kelly (2021).
